# Supplementary material for: Olivar: towards automated variant aware primer design for multiplex tiled amplicon sequencing of pathogens
Source: Nat Commun. 2024 Jul 26;15:6306. doi: 10.1038/s41467-024-49957-9 (PMC11282221; doi:10.1038/s41467-024-49957-9)
Supplement: Supplementary file 1 — Supplementary Information [file 41467_2024_49957_MOESM1_ESM.pdf]

Olivar: automated variant aware primer design for multiplex tiled amplicon sequencing

Michael X. Wang[1], Esther G. Lou[2], Nicolae Sapoval[3], Eddie Kim[3], Prashant Kalvapalle[2], Bryce Kille[3], R. A. Leo Elworth[3], Yunxi Liu[3], Yilei Fu[3], Lauren B. Stadler[2,*], and Todd J. Treangen[3,*]

[*]lauren.stadler@rice.edu, treangen@rice.edu
[1]Department of Bioengineering, Rice University, Houston, 77030, USA
[2]Department of Civil and Environmental Engineering, Rice University, Houston, 77005, USA
[3]Department of Computer Science, Rice University, Houston, 77005, USA

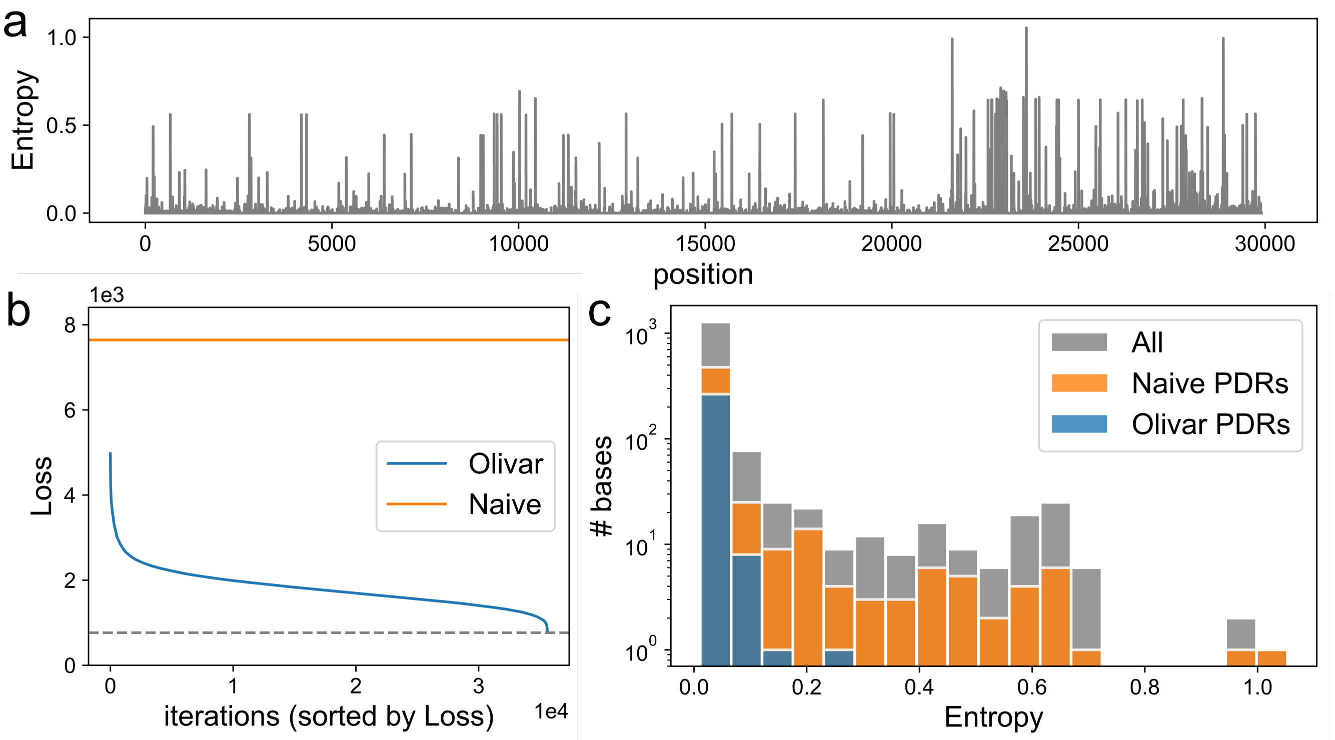

Figure S1. Optimization of PDRs with nucleotide entropy provided by the Nextstrain SARS-CoV-2 database. (a) Shannon entropy at each base position, provided by Nextstrain. Details about entropy calculation can be found in Methods. Entropy larger than 0.01 (1,517 bases) is input to Olivar as SNP frequency. **(b)** 35,584 PDR sets are generated by Olivar and the optimal PDR set with minimal Loss of 765.4 is chosen (dashed line). A randomly generated naive set of PDRs has a Loss of 7644.0. Optimization was run on a personal computer (2.4GHz 8-Core CPU) in 20 minutes wall clock, with peak memory usage less than 500MB. **(c)** Histogram of entropy of bases. Of the 1,517 bases with entropy greater than 0.01 (gray), 275 overlap with Olivar PDRs (blue), while 560 overlap with naive PDRs (orange).

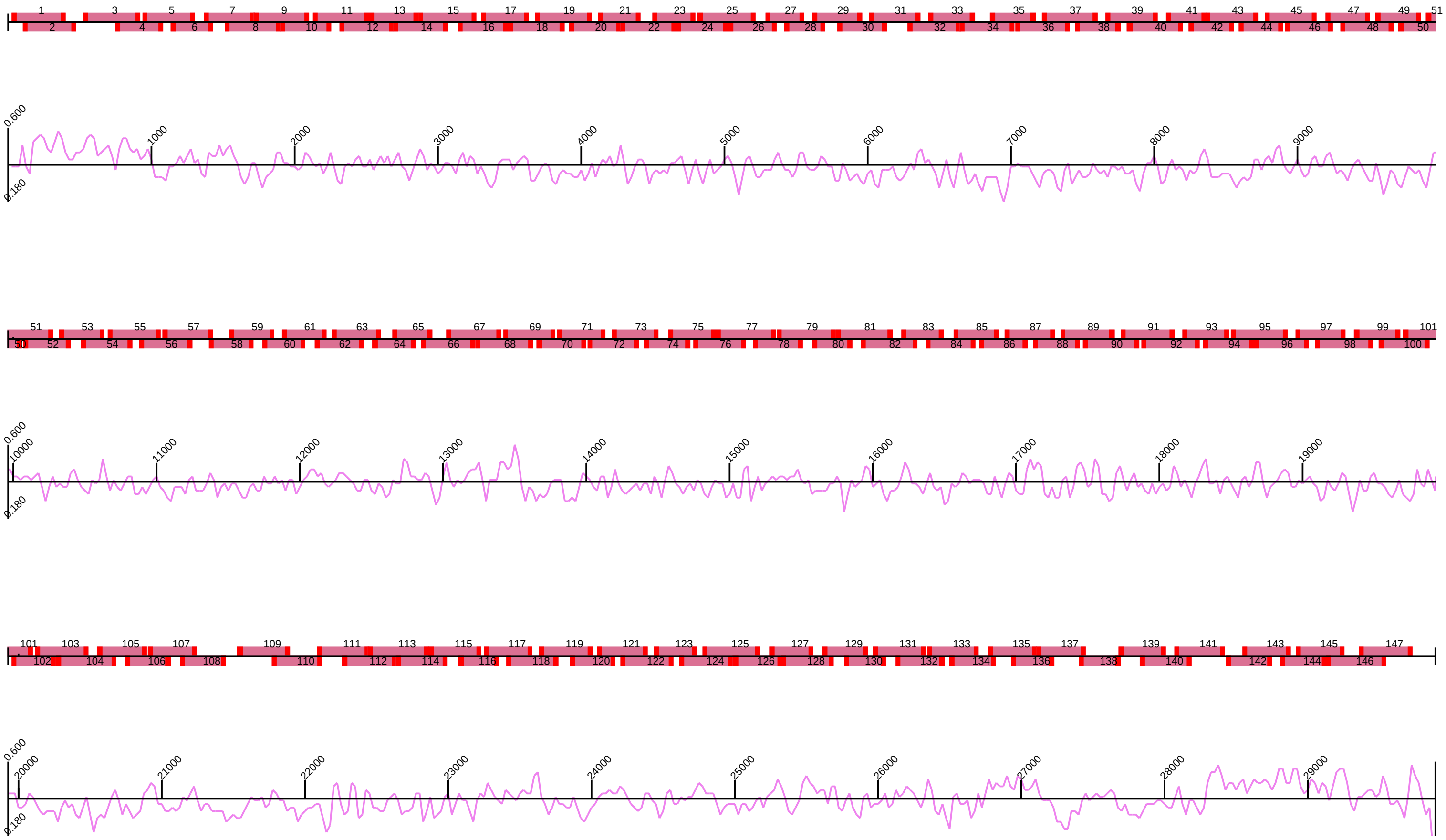

Figure S2. Location of amplicons designed by PrimalScheme, generated with the PrimalSheme software. 10 gaps are found in the PrimalScheme design: 448 to 553 (106bp), 9221 to 9225 (5bp), 11368 to 11393 (26bp), 12539 to 12539 (1bp), 21420 to 21561 (142bp), 22093 to 22113 (21bp), 26795 to 26799 (5bp), 27423 to 27431 (9bp), 27663 to 27710 (48bp) and 28395 to 28458 (64bp).

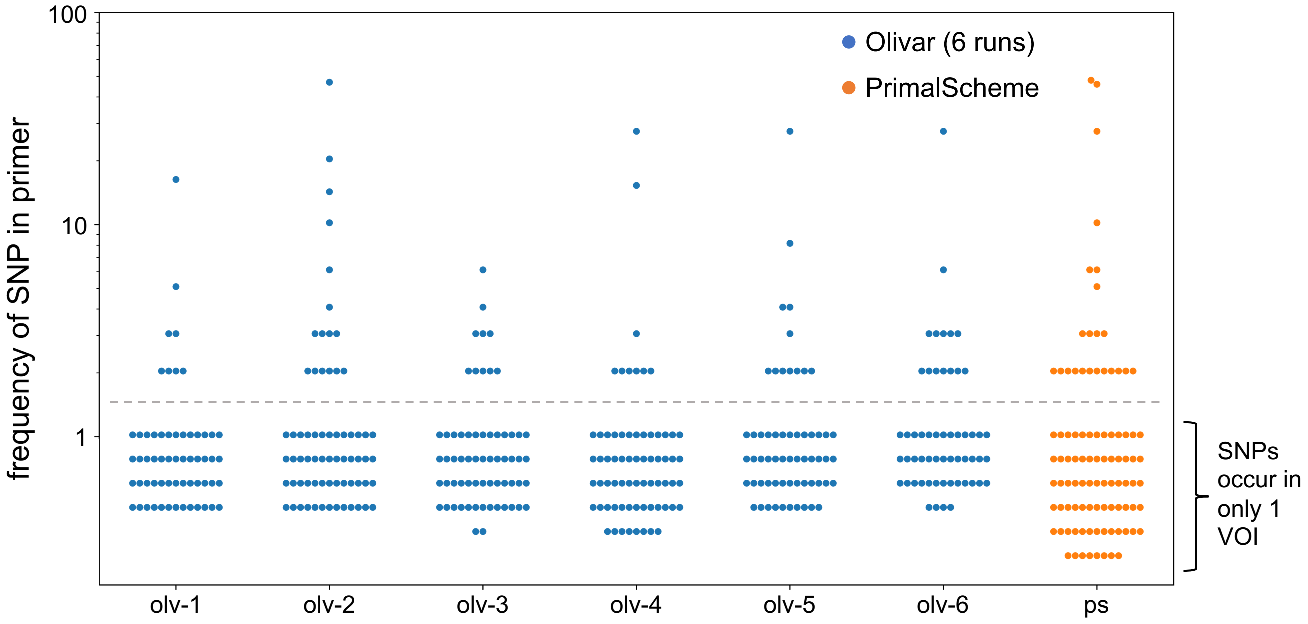

Figure S3. Frequencies of the SNPs that overlap with primers. 996 SNPs are called from 98 variants of interest (VOI) from GISAID, including substitutions, insertions and deletions. Note that data points below the gray dashed line are SNPs that occur in only 1 VOI. Olivar was run 6 times with 6 consecutive random seeds. More details can be found in Methods.

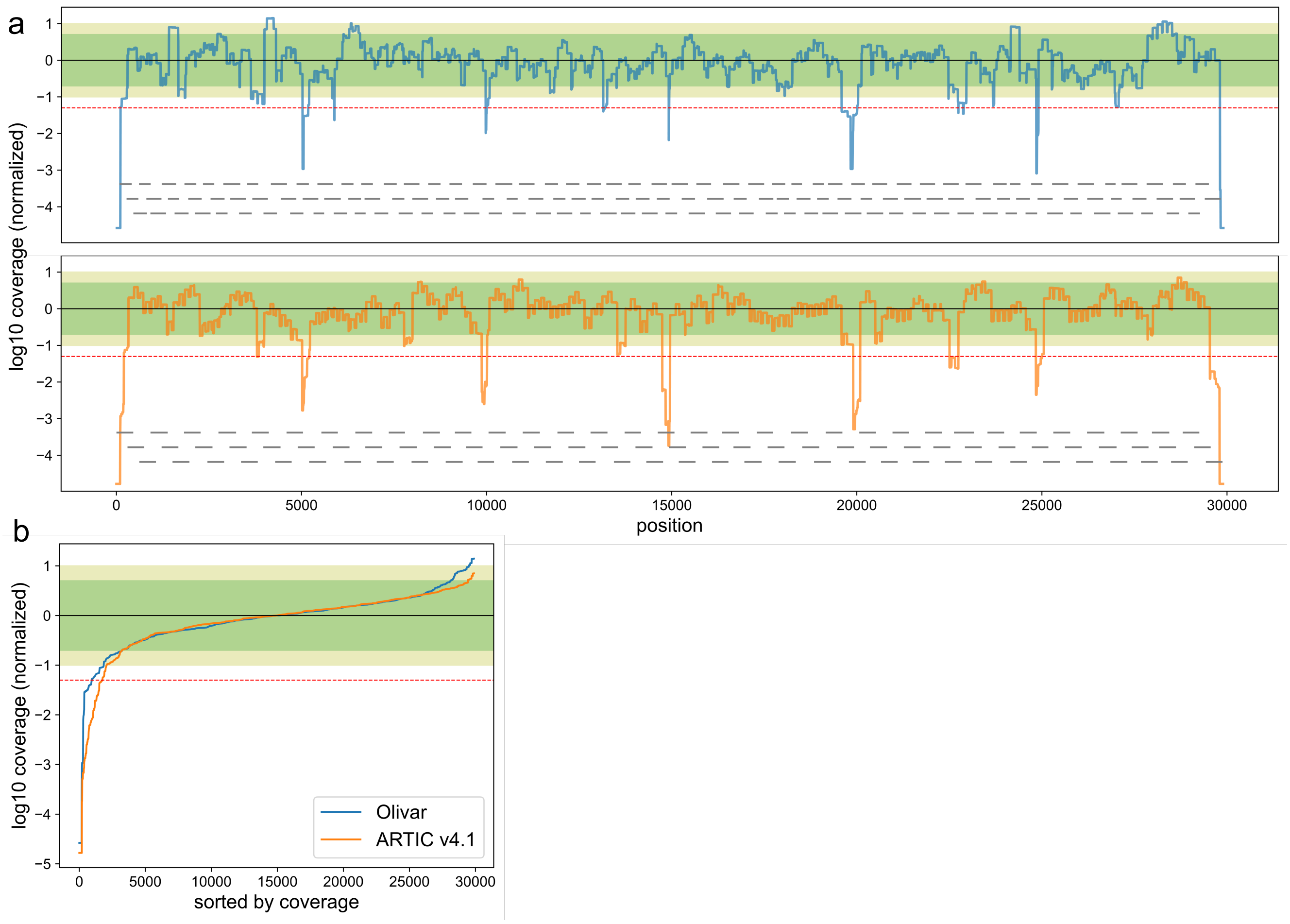

Figure S4. SARS-CoV-2 whole genome coverage of both Olivar (blue) and ARTIC v4.1 (orange) primers. Figures showing results from one wastewater sample (Ct=18, replicate 1). **(a)** log10 coverage of each base. Coverage is normalized by median coverage of all bases. Gray lines represent location of amplicons. **(b)** Sorted log10 coverage of each base. Black solid line represents the median coverage, green shade represents 0.2× to 5× median coverage (Olivar: 83.2% bases, ARTIC v4.1: 87.6% bases), olive shade represents 0.1× to 10× coverage (Olivar: 92.5% bases, ARTIC v4.1: 93.1% bases), red dashed line represents 0.05× median coverage (Olivar: 3.2% bases less than 0.05×, ARTIC v4.1: 5.9% bases less than 0.05×).

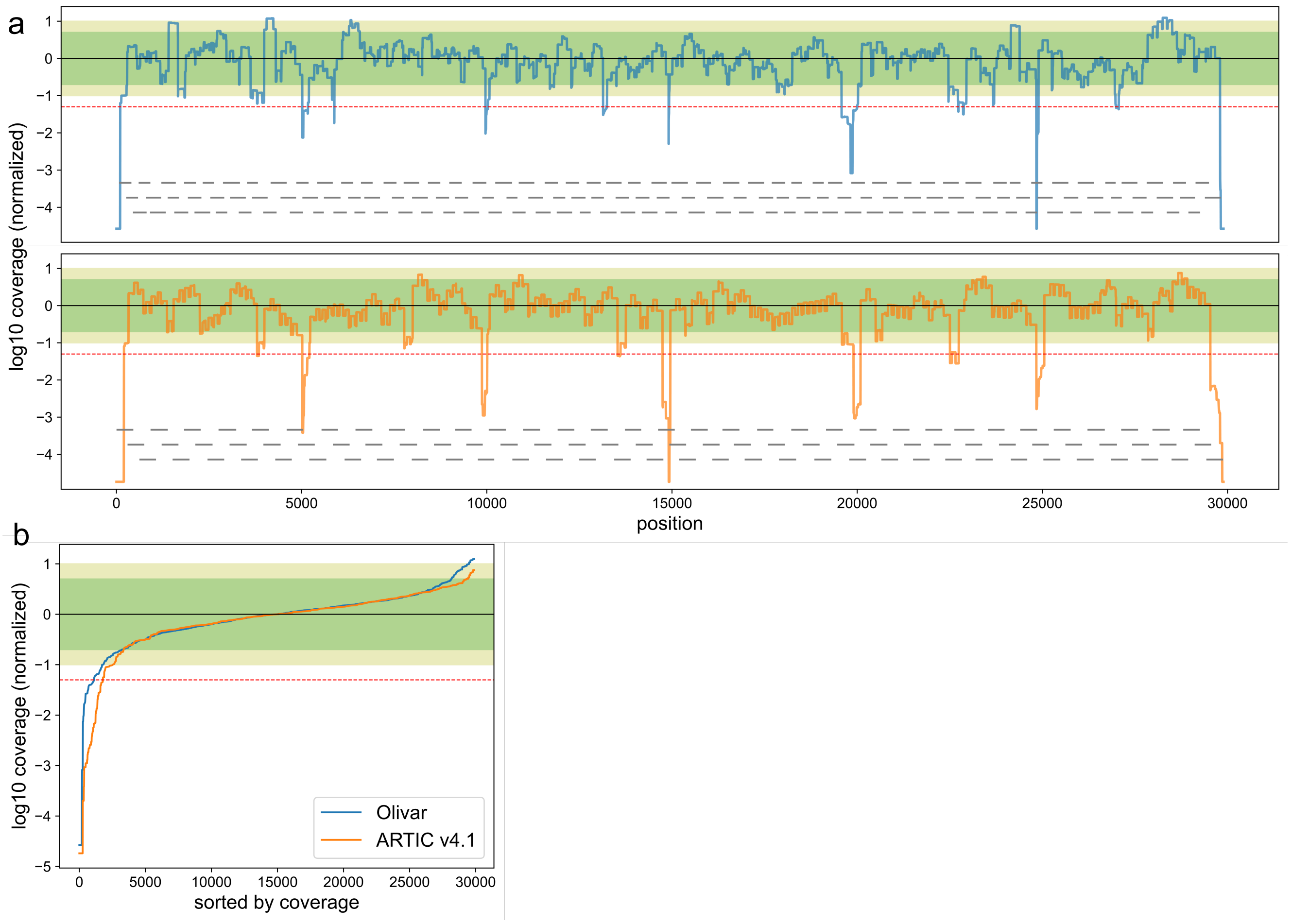

Figure S5. **SARS-CoV-2 whole genome coverage of both Olivar (blue) and ARTIC v4.1 (orange) primers. Figures showing results from one wastewater sample (Ct=18, replicate 2). (a)** log10 coverage of each base. Coverage is normalized by median coverage of all bases. Gray lines represent location of amplicons. **(b)** Sorted log10 coverage of each base. Black solid line represents the median coverage, green shade represents 0.2× to 5× median coverage (Olivar: 83.3% bases, ARTIC v4.1: 86.8% bases), olive shade represents 0.1× to 10× coverage (Olivar: 92.7% bases, ARTIC v4.1: 91.5% bases), red dashed line represents 0.05× median coverage (Olivar: 3.7% bases less than 0.05×, ARTIC v4.1: 6.0% bases less than 0.05×).

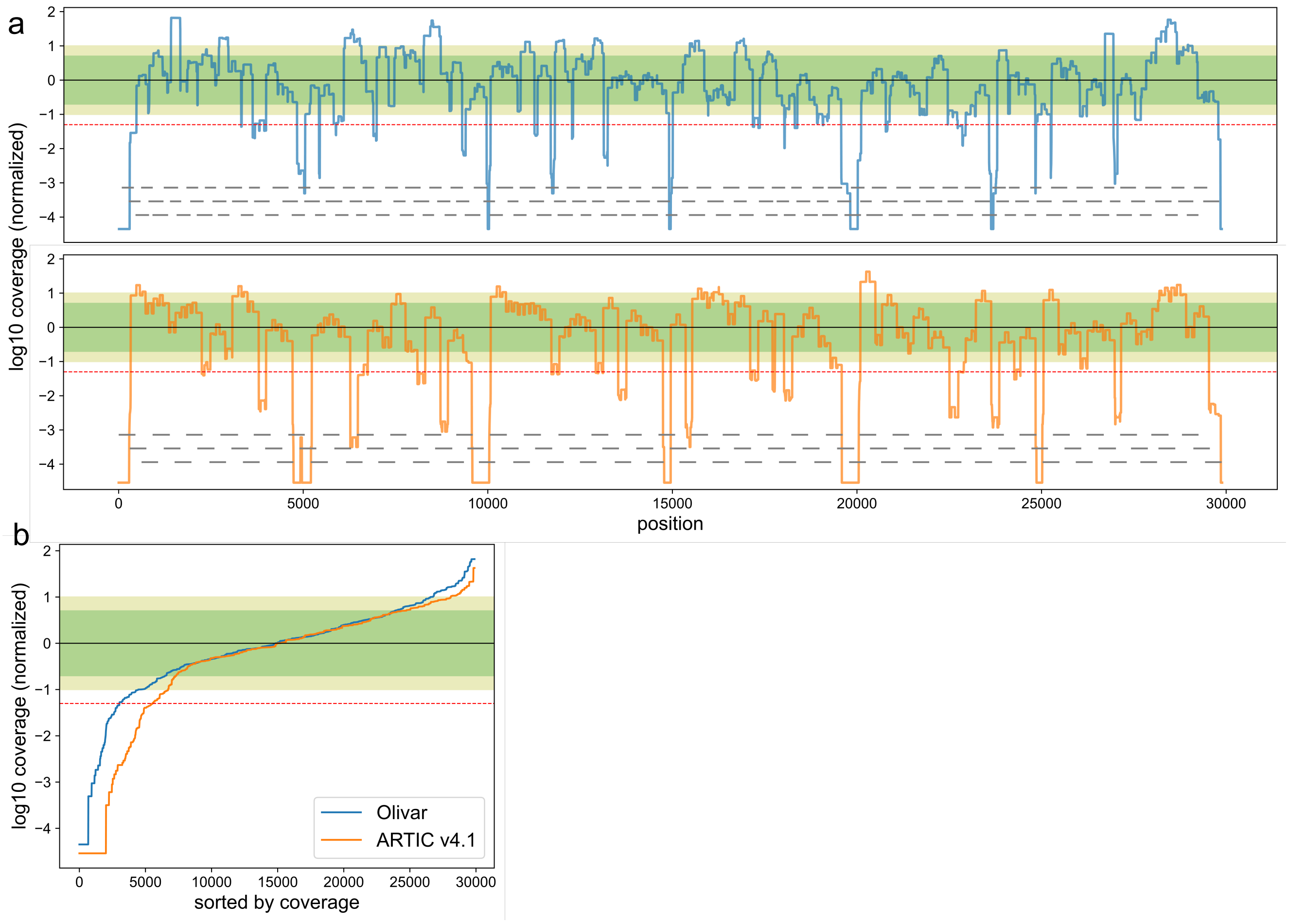

Figure S6. SARS-CoV-2 whole genome coverage of both Olivar (blue) and ARTIC v4.1 (orange) primers. Figures showing results from one wastewater sample (Ct=35, replicate 1). **(a)** log10 coverage of each base. Coverage is normalized by median coverage of all bases. Gray lines represent location of amplicons. **(b)** Sorted log10 coverage of each base. Black solid line represents the median coverage, green shade represents 0.2× to 5× median coverage (Olivar: 57.9% bases, ARTIC v4.1: 56.8% bases), olive shade represents 0.1× to 10× coverage (Olivar: 73.4% bases, ARTIC v4.1: 71.5% bases), red dashed line represents 0.05× median coverage (Olivar: 10.1% bases less than 0.05×, ARTIC v4.1: 18.5% bases less than 0.05×).

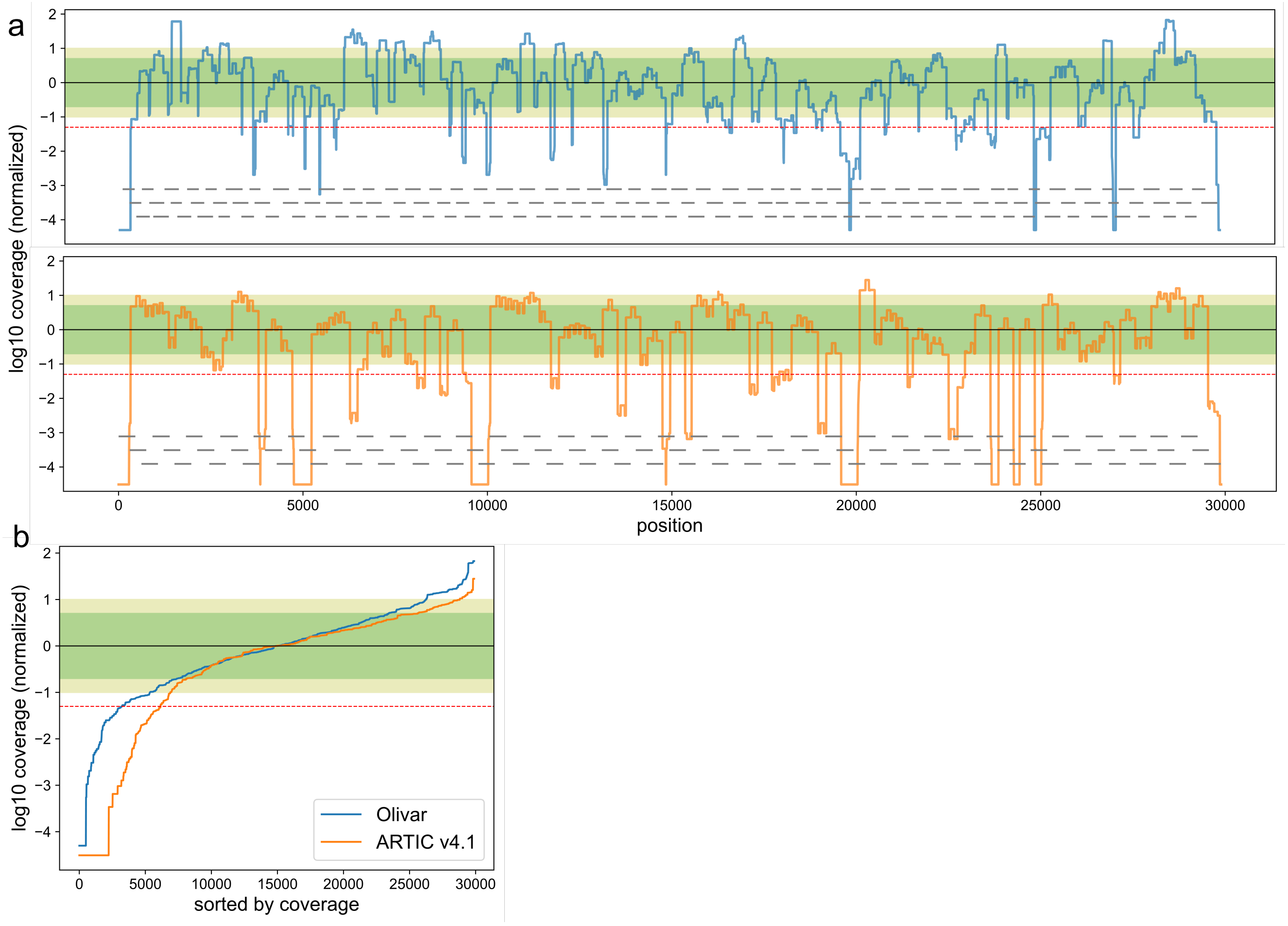

Figure S7. SARS-CoV-2 whole genome coverage of both Olivar (blue) and ARTIC v4.1 (orange) primers. Figures showing results from one wastewater sample (Ct=35, replicate 2). **(a)** log10 coverage of each base. Coverage is normalized by median coverage of all bases. Gray lines represent location of amplicons. **(b)** Sorted log10 coverage of each base. Black solid line represents the median coverage, green shade represents 0.2× to 5× median coverage (Olivar: 53.3% bases, ARTIC v4.1: 58.4% bases), olive shade represents 0.1× to 10× coverage (Olivar: 69.8% bases, ARTIC v4.1: 73.3% bases), red dashed line represents 0.05× median coverage (Olivar: 10.7% bases less than 0.05×, ARTIC v4.1: 20.6% bases less than 0.05×).

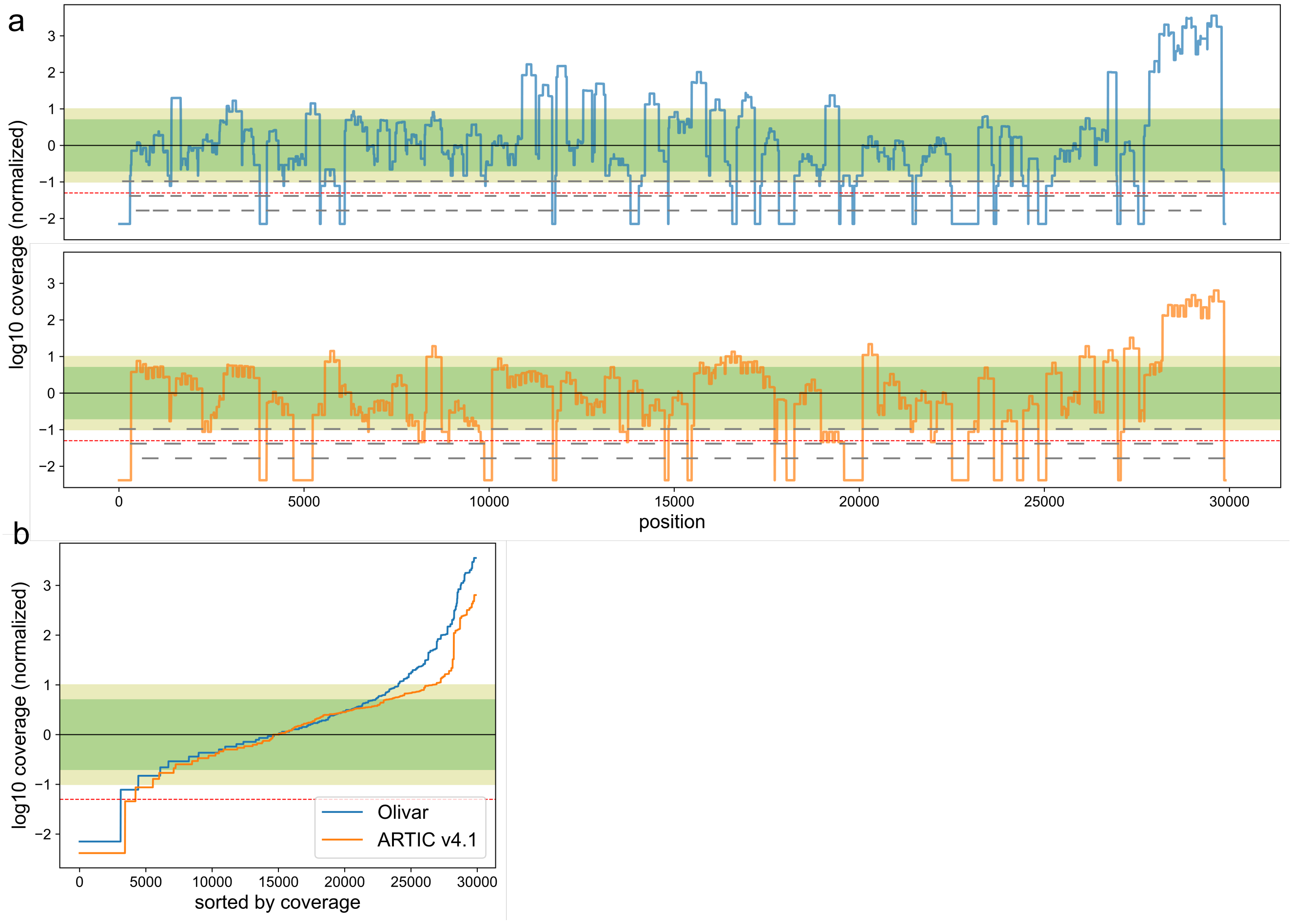

Figure S8. SARS-CoV-2 whole genome coverage of both Olivar (blue) and ARTIC v4.1 (orange) primers. Figures showing results from one wastewater sample (site: CB, Aug. 08, 2022). (a) log10 coverage of each base. Coverage is normalized by median coverage of all bases. Gray lines represent location of amplicons. **(b)** Sorted log10 coverage of each base. Black solid line represents the median coverage, green shade represents $0.2\times$ to $5\times$ median coverage (Olivar: 53.7% bases, ARTIC v4.1: 53.3% bases), olive shade represents $0.1\times$ to $10\times$ coverage (Olivar: 65.6% bases, ARTIC v4.1: 70.5% bases), red dashed line represents $0.05\times$ median coverage (Olivar: 10.4% bases less than $0.05\times$, ARTIC v4.1: 14.1% bases less than $0.05\times$).

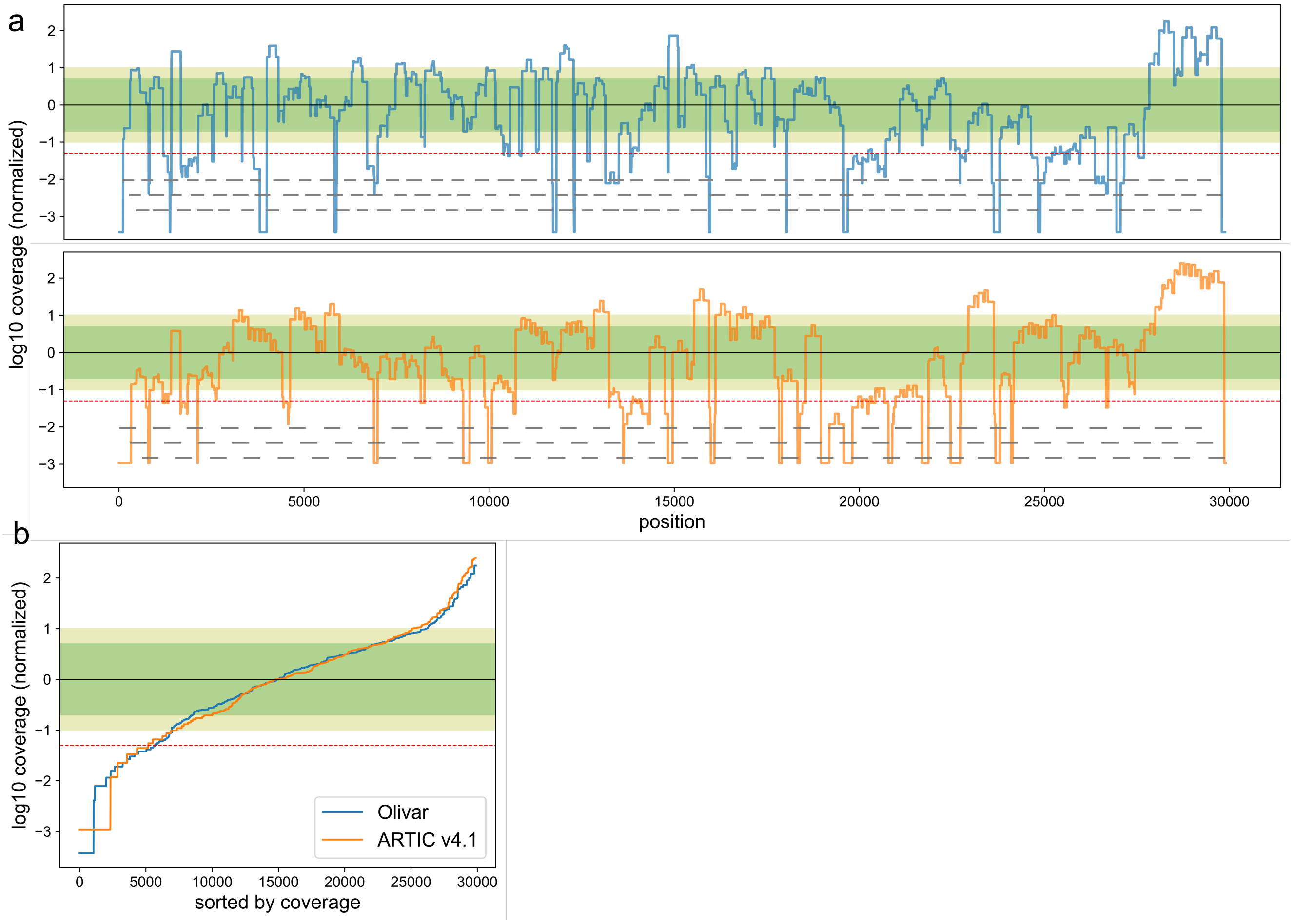

Figure S9. **SARS-CoV-2 whole genome coverage of both Olivar (blue) and ARTIC v4.1 (orange) primers. Figures showing results from one wastewater sample (site: KB, Aug. 08, 2022).** **(a)** log10 coverage of each base. Coverage is normalized by median coverage of all bases. Gray lines represent location of amplicons. **(b)** Sorted log10 coverage of each base. Black solid line represents the median coverage, green shade represents 0.2× to 5× median coverage (Olivar: 46.3% bases, ARTIC v4.1: 42.6% bases), olive shade represents 0.1× to 10× coverage (Olivar: 64.1% bases, ARTIC v4.1: 59.3% bases), red dashed line represents 0.05× median coverage (Olivar: 19.1% bases less than 0.05×, ARTIC v4.1: 17.3% bases less than 0.05×).

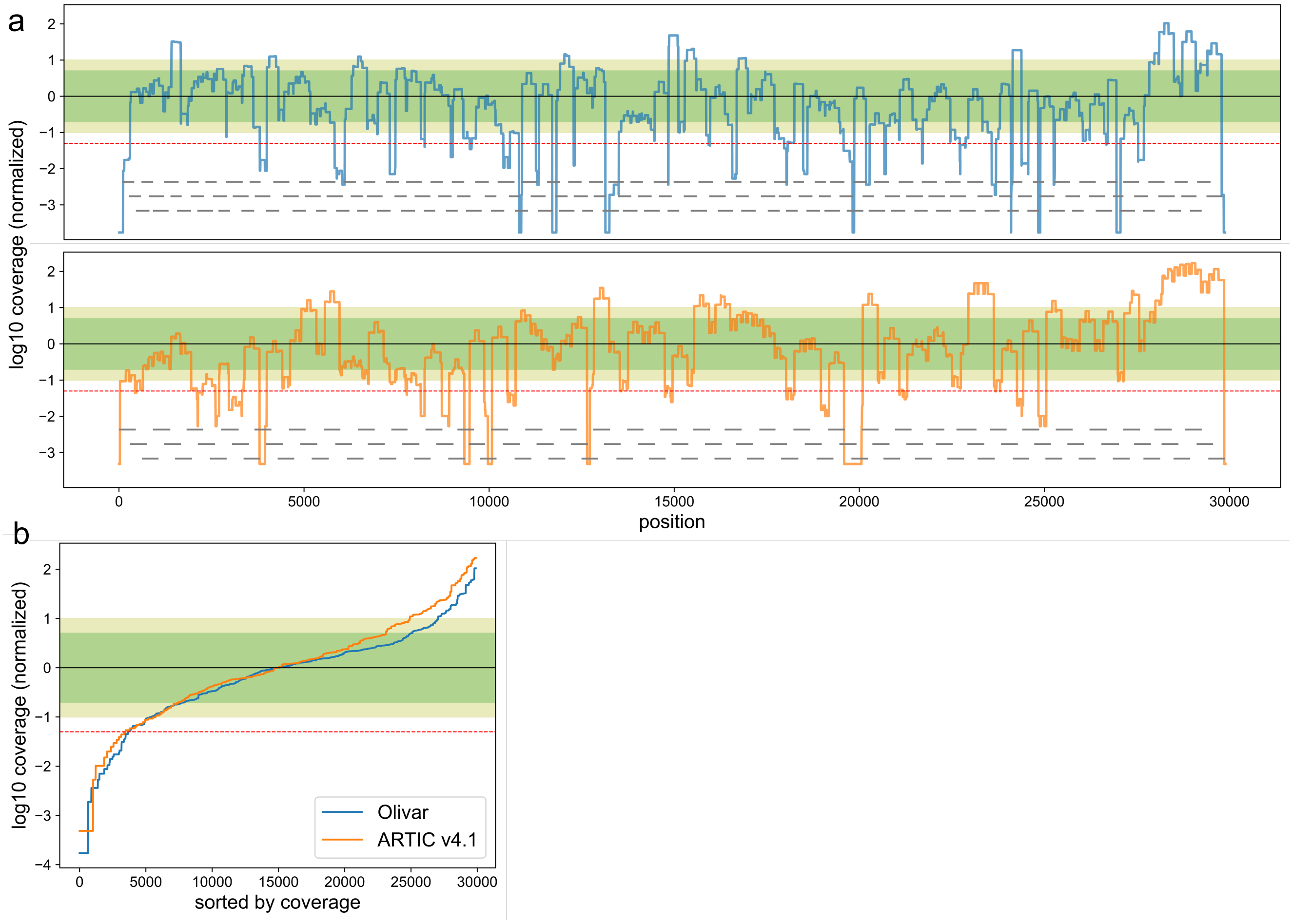

Figure S10. SARS-CoV-2 whole genome coverage of both Olivar (blue) and ARTIC v4.1 (orange) primers. Figures showing results from one wastewater sample (site: KB, Aug. 15, 2022). (a) log10 coverage of each base. Coverage is normalized by median coverage of all bases. Gray lines represent location of amplicons. **(b)** Sorted log10 coverage of each base. Black solid line represents the median coverage, green shade represents 0.2× to 5× median coverage (Olivar: 56.9% bases, ARTIC v4.1: 52.0% bases), olive shade represents 0.1× to 10× coverage (Olivar: 72.4% bases, ARTIC v4.1: 64.2% bases), red dashed line represents 0.05× median coverage (Olivar: 12.4% bases less than 0.05×, ARTIC v4.1: 11.6% bases less than 0.05×).

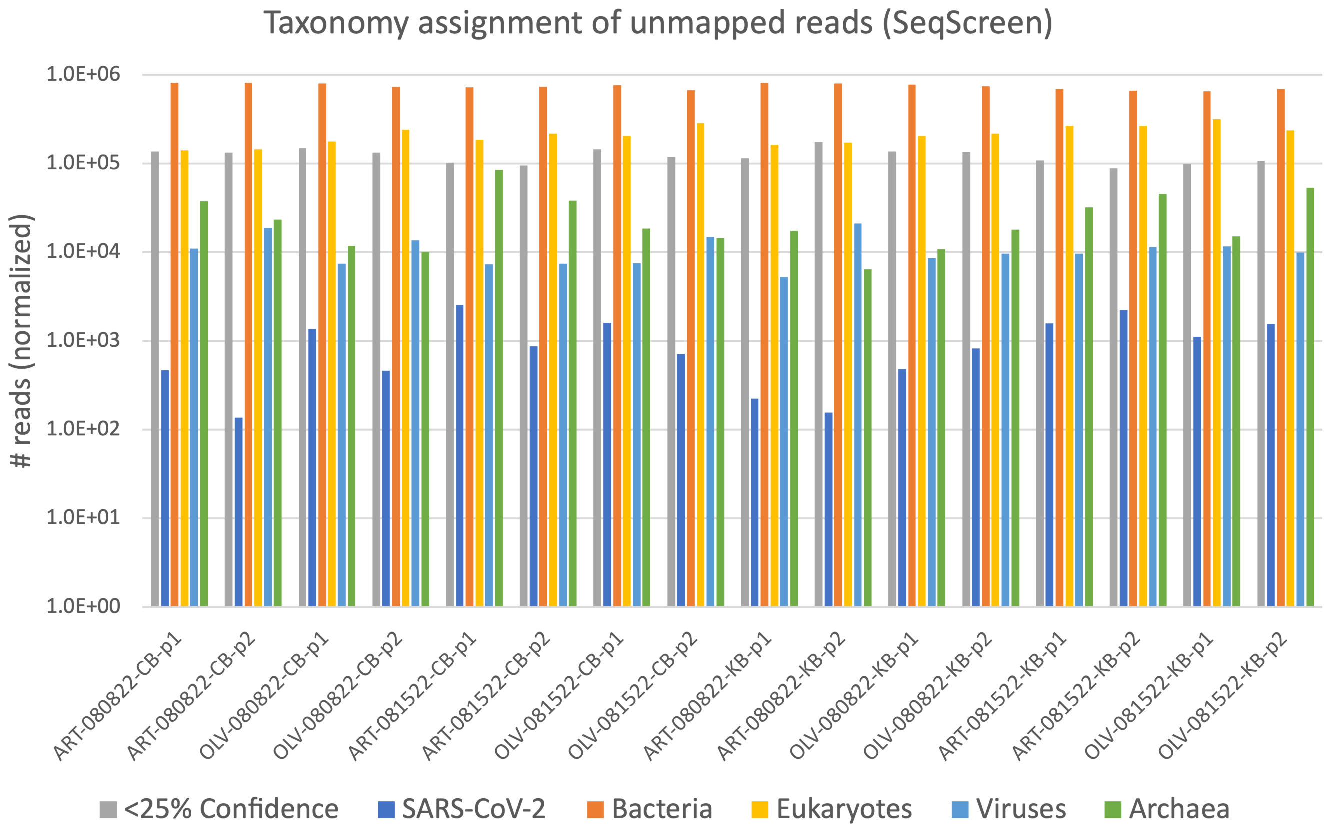

Figure S11. SeqScreen taxonomy assignment of unmapped reads of wastewater samples. Total unmapped reads of each sample is normalized to 1 million reads. On average 87.6% of unmapped reads are assigned with ≥25% confidence. Of those reads, 0.102% assigned to SARS-CoV-2, 74.3% assigned to bacteria, 21.6% assigned to eukaryotes, 2.74% assigned to archaea, 1.10% assigned to viruses. Sample ART-081522-CB-p1 has the highest fraction of 0.253% reads assigned to SARS-CoV-2. More details can be found in the Methods section.

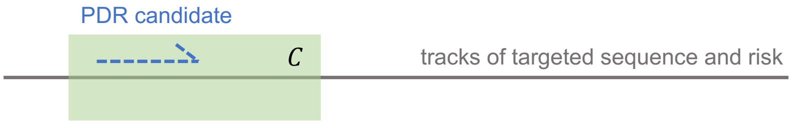

Figure S12. Described in Methods, Generation of one PDR.

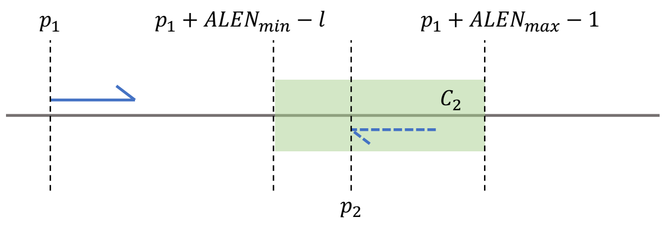

Figure S13. Described in Methods, Generation of a set of PDRs.

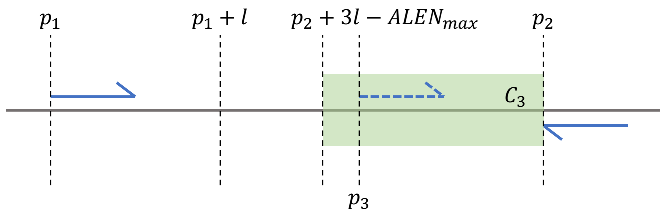

Figure S14. Described in Methods, Generation of a set of PDRs.

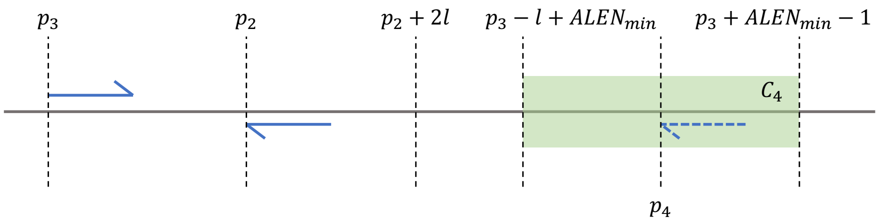

Figure S15. Described in Methods, Generation of a set of PDRs.

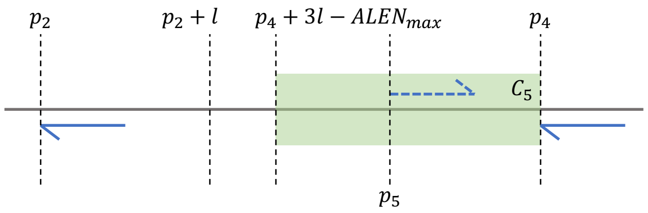

Figure S16. Described in Methods, Generation of a set of PDRs.

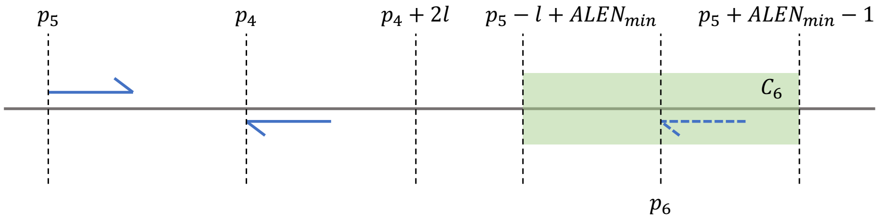

Figure S17. Described in Methods, Generation of a set of PDRs.
