# Supplementary material for: Olivar: towards automated variant aware primer design for multiplex tiled amplicon sequencing of pathogens
Source: Nat Commun. 2024 Jul 26;15:6306. doi: 10.1038/s41467-024-49957-9 (PMC11282221; doi:10.1038/s41467-024-49957-9)
Supplement: Supplementary file 3 — Description of Additional Supplementary Files [file 41467_2024_49957_MOESM3_ESM.pdf]

Description of Additional Supplementary Files

File Name: Supplementary Data 1-13

Description: This Excel file contains 13 sheets, with descriptions below.

Supplementary Data 1: Shannon's entropy for each base of the SARS-CoV-2 reference genome, calculated by NextStrain.

Supplementary Data 2: SNPs of Delta and Omicron variants, generated with Variant Database.

Supplementary Data 3 and 4: Olivar primers used for in-silico comparison and sequencing.

Supplementary Data 5 and 6: PrimalScheme primer used for in-silico comparison.

Supplementary Data 7: 98 variants of interest (VOI) of SARS-CoV-2 from GISAID

Supplementary Data 8: SNPs called from the MSA of 98 VOIs, including substitutions, insertions and deletions.

Supplementary Data 9 and 10: ARTIC v4.1 primers used for sequencing.

Supplementary Data 11: Instructions of preparing the ARTIC v4.1 primer pools.

Supplementary Data 12 and 13: Mapping rates and coverage uniformity calculated from sequencing libraries prepared with Olivar primers or ARTIC v4.1 primers.
